# Knockdown *SENP1* Suppressed the Angiogenic Potential of Mesenchymal Stem Cells by Impacting CXCR4-Regulated MRTF-A SUMOylation and CCN1 Expression

**DOI:** 10.3390/biomedicines11030914

**Published:** 2023-03-15

**Authors:** Rui Zhang, Qingxi Liu, Cuicui Lyu, Xing Gao, Wenjian Ma

**Affiliations:** 1 Department of Hematology, Tianjin First Central Hospital, Nankai University, Tianjin 300192, China; 2 State Key Laboratory of Medicinal Chemical Biology, College of Life Sciences, Nankai University, Tianjin 300071, China; 3 Department of Chemical and Biological Engineering, Qilu Institute of Technology, Jinan 250200, China; 4 College of Biotechnology, Tianjin University of Science and Technology, Tianjin 300457, China

**Keywords:** human mesenchymal stem cells (hMSCs), SENP1, CXCR4, CCN1, SUMOylation, MRTF-A, P300

## Abstract

The angiogenic potential of mesenchymal stem cells (MSCs) is critical for adult vascular regeneration and repair, which is regulated by various growth factors and cytokines. In the current study, we report that knockdown SUMO-specific peptidase 1 (SENP1) stimulated the SUMOylation of MRTF-A and prevented its translocation into the nucleus, leading to downregulation of the cytokine and angiogenic factor CCN1, which significantly impacted MSC-mediated angiogenesis and cell migration. Further studies showed that *SENP1* knockdown also suppressed the expression of a chemokine receptor CXCR4, and overexpression of CXCR4 could partially abrogate MRTF-A SUMOylation and reestablish the CCN1 level. Mutation analysis confirmed that SUMOylation occurred on three lysine residues (Lys-499, Lys-576, and Lys-624) of MRTF-A. In addition, *SENP1* knockdown abolished the synergistic co-activation of CCN1 between MRTF-A and histone acetyltransferase p300 by suppressing acetylation on histone3K9, histone3K14, and histone4. These results revealed an important signaling pathway to regulate MSC differentiation and angiogenesis by MRTF-A SUMOylation involving cytokine/chemokine activities mediated by CCN1 and CXCR4, which may potentially impact a variety of cellular processes such as revascularization, wound healing, and progression of cancer.

## 1. Introduction

Mesenchymal stem cells (MSCs) retain considerable potential for adult vascular repair and regeneration therapies [1]. Through differentiation and paracrine effects, MSCs can facilitate angiogenesis and new blood vessel formation [2]. Particularly, MSCs have been demonstrated that can enhance arteriogenesis, one of the most powerful revascularization mechanisms in adults initiated under various stress conditions [3].

Cytokines and growth factors play a key role in driving the differentiation of MSCs into specific tissue types by interacting with cell surface receptors to trigger downstream signaling pathways [4]. For example, the differentiation of human MSCs (hMSCs) into arterial cells can be induced by a high concentration of endothelial growth factor (VEGF), a key cytokine involved in the angiogenic process, via serum response factor (SRF)-dependent gene transactivation [5,6]. SRF is a transcription factor playing a key role in the transduction of mechanical signals from the cytoplasm or extracellular environment to the nucleus by controlling multiple downstream genes [7]. It controls cytoskeletal gene expression and cell migration and therefore is critically required for VEGF-induced angiogenesis.

Previous studies suggest that myocardin-related transcription factor (MRTF-A) plays important and dynamic roles during this process [8]. MRTF-A is a transcriptional coactivator of the SRF, which forms a complex with SRF to prevent it from binding to the CArG box promoter element of target genes [9,10]. MRTF-A is normally sequestered in the cytoplasm by binding to G-actin. When G-actin is occupied upon activation of the Rho signaling pathway and actin polymerization, free MRTF-A is then translocated to the nucleus and activates SRF [11,12]. The control of SRF activity by MRTF thus provides an important regulation for angiogenesis and related cellular functions mediated through SRF signaling pathways.

MRTFs are highly regulated to shuttle into the nucleus and exert their functions. Besides being regulated through MRTF-actin interactions, posttranslational modifications such as phosphorylation have also been shown as important regulatory mechanisms via Rho- and ERK-dependent pathways [13]. It has also been reported that MRTF-A could interact with small ubiquitin-related modification 1 (SUMO-1) in vitro based on a yeast two-hybrid screen [14]. However, the impacts of SUMOylation on the cellular function of MRTF-A are largely unknown.

SUMOylation is a dynamic process regulated by a family of SUMO-specific proteases called sentrin-specific protease (SENP), which reversely detaches SUMO molecules from the target proteins [15,16]. Among the six members of the SENP family, it was reported that SENP1 plays a critical role in coordinating developmental angiogenesis by affecting the SUMOylation of VEGFR2 and NOTCH1 [17,18].

The differentiation and angiogenesis of MSC are regulated by a complex signaling network with the possible involvement of multiple cytokines/chemokines. The CCN1 Protein of the cellular communication network (CCN) family is known to play crucial roles in angiogenesis, which not only interacts with various cytokines but also serves as an important proinflammatory cytokine in rheumatoid arthritis (RA) [19,20,21]. There was also evidence suggesting that the C-X-C receptor type 4 (CXCR4), a chemokine receptor for stromal cell-derived factor 1 (SDF-1), also plays crucial roles in angiogenesis and tissue recovery [22,23]. CXCR4 expression can be upregulated by hypoxia and angiogenic factors such as VEGF [24]. However, the connections between MRTF-A, CCN1, and CXCR4 in angiogenesis are yet to be fully elucidated.

To further understand the regulation network of MSC differentiation and artery-related angiogenesis, the current study investigated the impacts of *SENP1* knockdown and the signaling events underlying the SUMOylation of MRTF-A in this process.

## 2. Materials and Methods

### 2.1. Cell Culture

Human bone marrow-derived MSCs (hBM-MSCs) were granted from Union Stem Cell and Gene Engineering Co. (Tianjin, China). hBM-MSCs were grown in ECs differentiation medium (EDM) (Lonza) supplemented with 50 ng/mL VEGF (PeproTech, Cranbury, NJ, USA), 5 ng/mL basic fibroblast growth factor (bFGF) (PeproTech), and 2% fetal bovine serum (FBS) (PAA) for 7 days. Human aortic endothelial cells (HAECs) were purchased from Tianjin Medical University, China. The cells were seeded in Dulbecco’s Modified Eagle Medium-F12 Mixture (DMEM-F12, GIBCO, New York, NY, USA), supplemented with 10% FBS (Hyclone, Logan, UT, USA), and incubated at 37 °C in a fully humidified atmosphere of 5% CO_2_ in the air.

### 2.2. CD31/KDR Double-Positive Cell Sorting and Flow Cytometry

A total of 1 × 10^6^ cells were resuspended in 200 μL PBS for 20 min on ice to block non-specific antibody binding, followed by the addition of respective fluorochrome-conjugated antibodies (CD31-FITC and KDR-TRITC, 1–1.5 μg per 1 × 10^6^ cells) as well as Isotype IgG and negative controls that do not require primary antibodies. The mixture was vortexed and incubated on ice in the dark. After an hour, 400 μL PBS was added to each staining reaction and vortexed. Then, centrifuge and the cell pellet were resuspended in 500 μL of PBS. The above cell suspension was subject to cell sorting using the cell sorter (Becton Dickinson Immunocytometry Systems, Mountain View, CA, USA). The sorted CD31/KDR double-positive cells were resuspended in EDM and in vitro expansion. Flow Cytometry analysis for CXCR4 was performed using rabbit monoclonal antibodies (Abcam, Waltham, MA, USA, ab124824). Cells were fixed with 4% paraformaldehyde. A goat anti-rabbit IgG (Alexa Fluor 488, ab150077) was used as the secondary antibody at 1/2000 dilution.

### 2.3. Quantitative Real-Time PCR (qRT-PCR)

Total RNA was extracted using TRIzol reagent (Invitrogen, Waltham, MA, USA). RNA was used as the template for RT using random primers and M-MLV reverse transcriptase. The Fast SYBR Green Master Mix (Applied Biosystems, Waltham, MA, USA) was used to detect the mRNA levels of the specific genes by Real-Time PCR (Biosystems StepOne, Foster City, CA, USA, Applied Biosystems). The thermocycling conditions were as follows: denaturation for 30 s at 95 °C, annealing for 45 s at 48–70 °C, and a final extension step of 30 s at 72 °C, 30–32 cycles. Error bars represent the mean ± SE of three independent experiments that were performed in triplicate. The primers used for the PCR experiments were listed in Supplementary Information.

### 2.4. Plasmids and Cell Transfection

For transfection experiments, the lentivirus was prepared according to the manufacturer’s instructions (GeneCopoeia, Rockville, MD, USA). After incubation for 6 h, the medium was replaced by DMEM-F12 supplemented with 10% FBS. Transfection was performed according to the manufacturer’s instructions. PCR, western blotting, and luciferase analysis assays were then performed as described below.

### 2.5. SENP1 Knockdown Cells in ECs Differentiation Medium (EDM)

Lentiviral particles PLKO.1 and PLKO.1-shSENP1 were constructed from addgene. The primers used for the amplification of shSENP1 were shown in Supplementary Table S1. Viruses were packaged by co-transfection with PLKO.1 and PLKO.1-shSENP1 into 293T cells. The supernatants containing viruses were collected 48 h after transfection. Then, lentivirus was centrifuged and resuspended for further transduction of hMSCs. Subsequently, hMSCs were transduced with the lentivirus in Opti-MEM and then were grown in an Ecs differentiation medium (EDM).

### 2.6. Uptake of Acetylated Low-Density Lipoprotein (DiI-Ac-LDL)

The cells of every experimental group were washed with PBS. Cells were fixed with 4% paraformaldehyde and 10 ug/mL DiI-Ac-LDL (Biomedical Technologies Inc., Stoughton, MA, USA) and incubated at 37 °C for 30 min. The samples were visualized using a laser scanning confocal microscope (Olympus, Tokyo, Japan).

### 2.7. Cell Migration Assay

hMSCs^shCtrl^ and hMSCs^shSENP1^ were grown in 6-well plates and wounded using a sterile pipette tip. The progress of migration was recorded immediately following injury, and photo-micrographs were taken at zero and 48 h.

### 2.8. Transwell Chamber Assay

hMSCs^shCtrl^ and hMSCs^shSENP1^ were seeded into the upper chamber of a transwell cell culture insert with 1.0 × 10^4^ cells in 200 µL of a 1% FBS-containing medium. The lower chamber was filled with 600 µL of medium containing 10% FBS. Twenty-four hours later, cells that had migrated to the lower side of the membrane were fixed in 4% paraformaldehyde and stained with DAPI. The migrated cells were counted and photographed in five fields of view (i.e., the upper, lower, left, right, and middle fields), which was performed in three independent experiments.

### 2.9. Immunocytochemistry Assay

The cells after treatment were fixed in 4% paraformaldehyde for 20 min and then blocked with normal goat serum for 30 min at room temperature. After incubation with rabbit anti-KDR (Abcam, ab2349) and anti-MRTF-A (Abcam, ab219981) in a humid chamber overnight, cells were incubated with appropriate secondary antibodies (FITC conjugated goat anti-rabbit IgG, Santa Cruz, CA, USA) for 30 min at 37 °C. After washing with PBS, the samples were observed under a confocal laser scanning microscope. DAPI stain (blue) highlights the total nuclei.

### 2.10. In Vitro Angiogenesis Assay

hMSCs^shCtrl^ and hMSCs^shSENP1^ were cultured in EDM for 7 days and capillary tube formation was induced using basement membrane-like material (EC Matrix TM; BD). Basement membrane-like material was diluted to 0.5–0.7 mg/mL in EDM. A total of 5 × 10^4^ cells were mixed in 300 μL Matrigel with 50 ng/mL VEGF in each well of a 24-well plate. The structures were photographed using a phase contrast microscope (Olympus) after 2 d. Total cord length was quantified using image-Pro Plus v4.5 software.

### 2.11. Western Blotting

Western immunoblotting was performed as described previously [3]. SDS-PAGE was used to separate the proteins and the membranes were subsequently incubated with different rabbit primary antibodies for CCN1 (Abcam, ab230947), SENP1 (Abcam, ab108981), CXCR4 (Abcam, ab124824), Hey2 (Abcam, ab86010), EphrinB2 (Abcam, ab131536), Dll4 (Abcam, ab176876), mouse anti-MRTF-A (Abcam, ab219981), and mouse anti-GAPDH (Santa Cruz, sc-166574) overnight at 4 °C. The secondary antibodies were IRDye-800-conjugated anti-mouse and anti-rabbit immunoglobulin G (Li-COR Biosciences, Lincoln, NE, USA) (1:200). Immunoreactivity was detected using an Odyssey Infrared Imaging System (Gene Company Ltd., Chai Wan, Hong Kong). GAPDH expression was used as an internal control. The relative quantification of protein expression was analyzed using ImageJ software (version 1.53).

### 2.12. Co-Immunoprecipitation

The lysates of hMSCs^shCtrl^ and hMSCs^shSENP1^ treated with EDM were collected. MRTF-A antibodies and Protein A agarose (Millipore, Burlington, MA, USA) were then used to precipitate MRTF-A from the whole cell lysate. The resulting mixture was washed, subjected to SDS-PAGE, transferred to nitrocellulose (NC) membranes, and probed with SUMO-specific antibodies to visualize SUMO-1-MRTF-A.

### 2.13. Luciferase Assay

Transfection reporter assays were performed in 24-well plates. Cells were harvested 24 h after transfection and luciferase activity was measured using the Dual luciferase assay system (Promega, Madison, WI, USA). Unless otherwise indicated, 100 ng of reporter and 400 ng of activator plasmids were used. Results were normalized by dividing the Firefly luciferase activity with the Renilla luciferase activity of the same sample. Each sample was examined in duplicate, and it was repeated in 3 different experiments.

### 2.14. Chromosomal Immunoprecipitation (ChIP) Assay

ChIP analysis was performed in HAECs co-transfected with MRTF-A, P300, and shSENP1 plasmids, using a commercially available Enzymatic Chromatin IP kit with magnetic beads (Cell Signalling Technology, Danvers, MA, USA). The proteins were cross-linked to DNA by formaldehyde at 2% concentration for 30 min at room temperature. The protein-DNA complexes were immunoprecipitated using primary antibodies against MRTF-A (1:200; Sigma-Aldrich; Merck KGaA, Darmstadt, Germany). The signals of the CCN1/MRTF-A/P300/shSENP1 promoter complexes were measured by PCR. The primers used for the amplification of *CCN1* were listed in Supplementary Information (Table S2).

### 2.15. Statistical Analysis

Data were expressed as mean ± SE, accompanied by the number of experiments performed independently, and analyzed by Student’s *t*-test. Differences at an alpha value of *p* < 0.05 were considered statistically significant differences between pairs or groups of data.

## 3. Results

### 3.1. SENP1 Is Involved in the Differentiation of hMSCs into Artery-Specific Endothelial Cells

**Figure 1:**
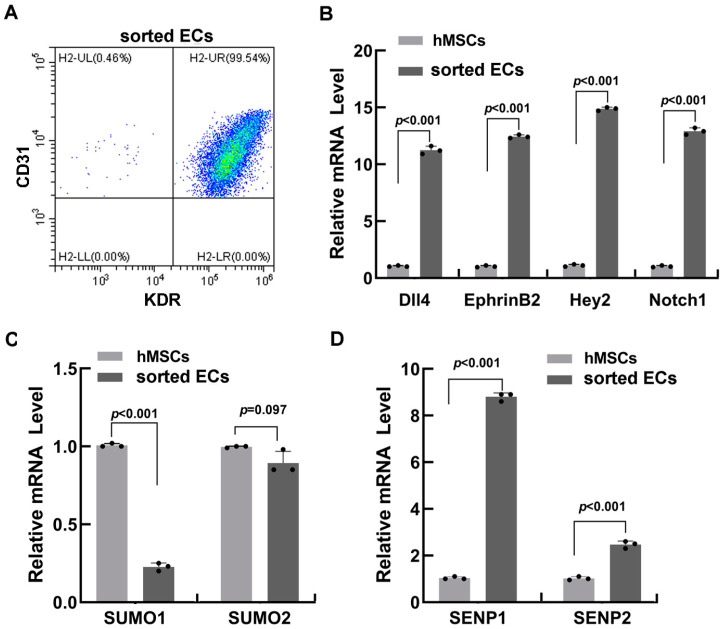
SENP1 participates in arterial-specific endothelial differentiation of hMSCs. (**A**) MSC-differentiated EC cells (CD31- and KDR-positive) were sorted at 7 days and cultured in EDM media with a purity of 99.54%. (**B**) Arterial-specific marker genes (*Dll4*, *EphrinB2*, *Hey2*, and *Notch1*) were examined in obtained ECs by qRT-PCR. (**C**,**D**) The expression changes of SUMO1-2 and SENP1-2 during endothelial differentiation were determined by qRT-PCR. Bar graphs are shown as the mean ± SD with individual points from three independent experiments (*p* values were indicated in the graph, individual data points were shown as scattered black dots).

To study whether post-translational modification by SUMO may be involved in regulating arterial-specific endothelial differentiation of hMSCs, we first determined the expression of SUMO1 and SUMO2, the two most distantly related SUMO family paralogues. Following induction of differentiation in EDM media, CD31- and KDR-positive cells were separated by magnetic bead sorting and flow cytometry at day 7 with a purity of 98.5% (Figure 1A). The obtained artery-specific ECs were confirmed based on the expression of arterial marker genes (*EphrinB2*, *Hey2*, *Dll4*, and *Notch1*) [25,26], which were significantly elevated as determined by RT-PCR (Figure 1B). Compared to hSMCs, the expression of SUMO1 in ECs was significantly reduced while SUMO2 was not much changed, indicating that SUMO1 may play a role in facilitating the differentiation toward the formation of artery-specific ECs (Figure 1C). In line with the suppression of SUMO1 transcription, the transcription of the deSUMOylating enzymes, SUMO-specific peptidase 1 (SENP1), and 2 (SENP2), was both significantly increased after differentiation (Figure 1D). The upregulation of SENP1 was more prevalent, that is over four-fold of SENP2, indicating that it may be a major modifier of SUMO1 level during differentiation.

### 3.2. SENP1 Stable Knockdown Suppresses Endothelial-Specific Differentiation of hMSCs and Exhibits Weaker Migration and Angiogenesis Potential

**Figure 2:**
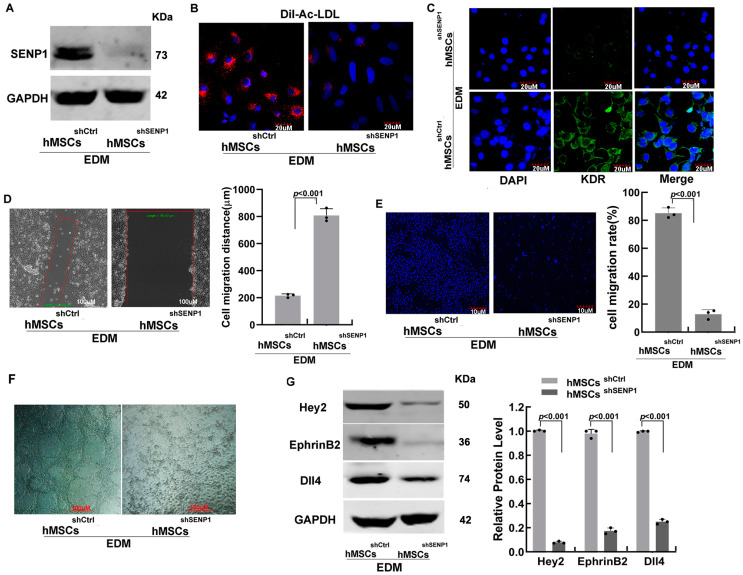
*SENP1* stable knockdown suppressed endothelial-specific differentiation of hMSCs and led to reduced migration and angiogenesis capacity. (**A**) Stable knockdown cell lines of the *SENP1* gene were generated by lentivirus-based RNAi. (**B**) DiI-Ac-LDL uptake analysis of hMSCs^shSENP1^ and hMSCs^shCtrl^. (**C**) Immunofluorescence detection of endothelial marker KDR in hMSCs^shSENP1^ and hMSCs^shCtrl^. (**D**) Cell migration determined by wound healing assay, and the migration rate was quantified by measuring the scratch gap width after 24 h of cell culture. (**E**) The invasion capacity of hMSCs^shSENP1^ and hMSCs^shCtrl^ was determined by transwell chamber assay, and the migration rate was quantified by measuring the percentage of cells that migrated across the membrane after 24 h of cell culture. (**F**) Angiogenesis determination by Matrigel based on the formation of capillary-like structures. (**G**) The protein expression of arterial-specific genes *Dll4*, *EphrinB2*, *Hey2*, and *Notch1* in hMSCs^shSENP1^ and hMSCs^shCtrl^ and quantification. Experiments were repeated three times, and quantitation is shown as the means ± SD from three independent experiments (*p* values were indicated in the graph, and individual data points were shown as scattered black dots).

To understand the role of SUMO posttranslational protein modification during hMSC differentiation, we generated *SENP1* stable knockdown hMSCs by lentivirus-based shRNA (Figure 2A). As determined by the DiI-Ac-LDL uptake assay based on the characteristic property of endothelial cells via their scavenger cell pathway of LDL metabolism, results showed that the SENP1-competent hMSCs acquired the ability to incorporate DiI-Ac-LDL following EDM-induced differentiation, whereas *SENP1*-knockdown hMSCs lost this capability (Figure 2B), giving the first indication that the differentiation toward ECs was inhibited. Further analysis of the endothelial-specific gene *KDR* also showed that it was significantly inhibited in hMSCs^shSENP1^ compared to WT hMSCs (Figure 2C). These results demonstrate that SENP1 is indispensable during the differentiation of hMSCs toward endothelial lineage. Interestingly, the knockdown of *SENP1* also showed suppressing effects on cell migration and angiogenesis. As determined by the gap-filling assay, the gap was significantly less filled in hMSCshSTC1 compared to control hMSCs after 48 h (Figure 2D). The inhibitory effect on cell migration was further confirmed by the transwell assay. As shown in Figure 2E, significant cell migration and invasion were observed in hMSCs^shCtrl^, whereas there was over four times reduction in the cell numbers migrating across the Transwell chamber membrane in hMSCs^shSENP1^. To assess angiogenesis, monolayer cell culture on the surface of 3D gels in three-dimensional in vitro assays was performed after induction with EDM for 48 h. While WT hMSCs can form vessel-like structures, it was not formed in *SENP1*-knockdown hMSCs (Figure 2F). In addition, the expression of arterial-specific genes *Dll4*, *EphrinB2*, *Hey2*, and *Notch1* were significantly inhibited in hMSCs^shSENP1^ (Figure 2G). Taken together, the above results demonstrated that SENP1 plays an important role not only in the differentiation of hMSCs toward artery-specific ECs but also in the obtaining of angiogenesis capacity.

### 3.3. SENP1 Knockdown Led to SUMO1 Modification of MRTF-A

**Figure 3:**
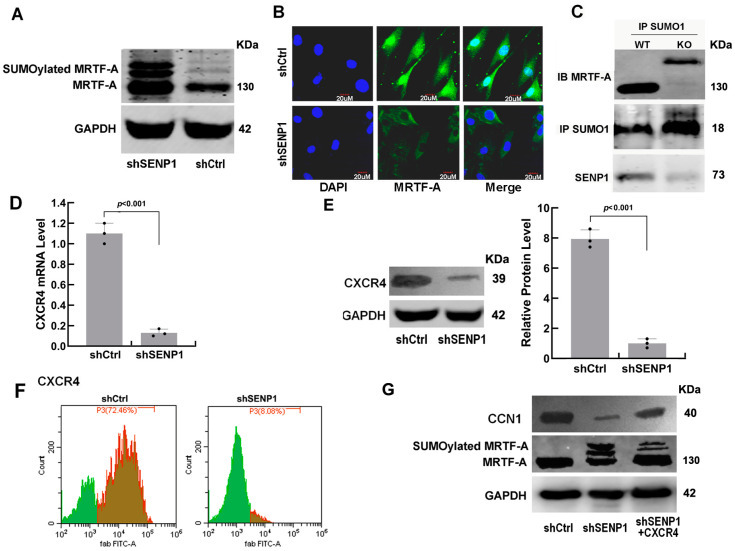
SUMO1 modification of MRTF-A after *SENP1* knockdown, and the impact of CXCR4. (**A**) MRTF-A SUMOylation analyzed by Western blotting in hMSCs^shSENP1^ and hMSCs^shCtrl^ after EDM-induced differentiation. (**B**) Immunofluorescence detection of MRTF-A cellular localization. (**C**) Co-immunoprecipitation analysis to determine the conjugation between MRTF-A and SUMO1. (**D**,**E**) The expression of CXCR4 in hMSCs^shSENP1^ and hMSCs^shCtrl^ determined by mRNA level and protein expression. (**F**) The expression of CXCR4 analyzed by flow cytometry, which was represented by a green peak for CXCR4- cells and a red-brown peak for CXCR4+ cells. (**G**) Over-expressing CXCR4 partially abrogated the impact of *SENP1* knockdown on MRTF-A SUMOylation and CCN1 expression. Quantitation is shown as the means ± SD (*p* values are as indicated, *n* = 3. Individual data points were shown as scattered black dots).

Considering that MRTF-A has been reported that can promote both artery-specific differentiation and tumor cell metastasis [27], we questioned whether the suppressed angiogenesis following *SENP1* knockdown may be mediated by its interaction with MRTF-A. SUMOylation of MRTF-A was determined by western blot in cell lysates using an antibody for MRTF-A. As shown in Figure 3A, the level of SUMOylation was evidently increased in hMSCs^shSENP1^ undergoing differentiation compared to that in the ECs differentiated from control WT hMSCs, which showed additional slower-migration bands as determined by western blotting. Further analysis by immunofluorescence imaging indicated that knockdown of *SENP1* suppressed the translocation of MRTF-A to the nucleus (Figure 3B). To confirm the slower-migrating protein bands were SUMO-1 conjugate, co-immunoprecipitation assays were performed. As shown in Figure 3C, the MRTF-A antibody identified a band that reacted with anti-SUMO1 in the hMSCs^shSENP1^ group. It is worth mentioning that the pull-down consisted of an antibody-MRTF-A complex with varying numbers of SUMO groups. Due to the large size of the complex, SUMOylated proteins were all detected as a single band. The above data demonstrated that *SENP1* knockdown significantly enhanced the SUMOylation of MRTF-A and prevented its translocation into the nucleus.

### 3.4. SENP1 Knockdown Suppressed the Expression of CXCR4, Which Was Causatively Related to SUMO1 Modification of MRTF-A and CCN1 Expression

In an attempt to screen for other factors that may be affected by *SENP1* knockdown, we identified that the expression of the chemokine receptor CXCR4 was significantly suppressed on both mRNA and protein levels as determined by RT-PCR and western blotting (Figure 3D,E). FACS analysis further confirmed that the percentage of CXCR4 positive cells was evidently reduced from ~70% in hMSCs^shCtrl^ to less than 10% in MSCs^shSENP1^ (Figure 3F). To further investigate whether CXCR4 suppression was causatively involved in the regulation of MRTF-A, we determined its impact on the SUMOylation of MRTF-A. As shown in Figure 3G, overexpression of CXCR4 in MSCs^shSENP1^ cells could partially inhibit MRTF-A SUMOylation level. In addition, CXCR4 overexpression also abrogated the suppression of CCN1 by MRTF-A SUMOylation. CCN1 is a key angiogenic factor that will be further discussed below. Furthermore, overexpression of CXCR4 in hMSCs^shSENP1^ evidently restored the cell invasion capacity (Supplementary Figure S1) and preliminary results showed that knockdown of SENP1 also suppressed the expression of SDF-1 mRNA level in hMSCs (Supplementary Figure S2). These data suggest that CXCR4 was likely a negative regulator of MRTF-A SUMOylation in addition to SENP1.

### 3.5. SUMOylation of MRTF-A Occurred on Three Lysine Residues and Played a Critical Role in Transactivating the Angiogenic Factor CCN1

**Figure 4:**
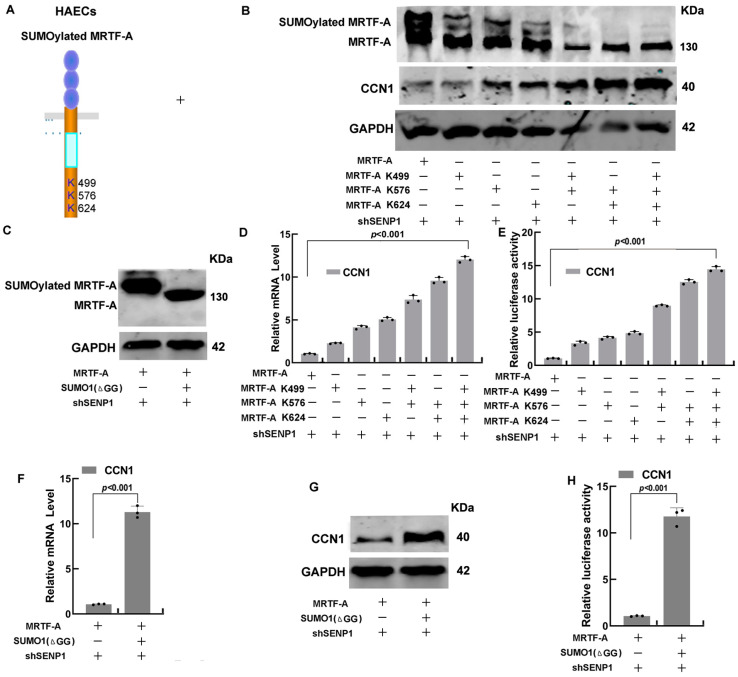
The correlation between CCN1 transactivation and MRTF-A SUMOylation. (**A**) The three putative SUMOylation sites of MRTF-A are located within amino acids 471-630. (**B**) Confirmation of SUMO-binding sites with specific point mutations (K499, K576, and K624) of MRTF-A together with shSENP1. The whole cell lysates were subjected to western immunoblotting with an anti-MRTF-A specific antibody. (**C**) SUMO-1 specific SUMOylation of MRTF-A was confirmed by transient transfection with conjugation-defective SUMO-1 (∆GG) HAECs. (**D**,**E**) Increased expression of CCN1 following disruption of SUMOylation by the transient introduction of K499/576/624R mutations examined by qRT-PCR and western blot. (**F**) Luciferase analysis of CCN1 activation in WT and lysin mutants of MRTF-A. (**G**,**H**) Abolishing SUMOylation with conjugation-defective SUMO-1 (∆GG) led to increased expression of CCN1 as examined by qRT-PCR and western blot, and transactivation as determined by luciferase assay. The signs ‘+’ means present and ‘−’ means absent for the conditions indicated in each graph. Quantitation is shown as the means ± SD (*p* values are as indicated, *n* = 3. Individual data points were shown as scattered black dots).

To investigate the acceptor sites for SUMOylation, a series of MRTF-A missense mutants on known lysine residues that can form SUMO conjugates were created (Figure 4A). The expression vectors of the MRTF-A lysine-substitution single mutants (K499R, K576R, and K624R), double mutants, or triple mutants were transfected into HAECs concomitant with ^shSENP1^, and their impacts on SUMOylation were tested. As shown in Figure 4B, the three MRTF-A single mutants could partially suppress the formation of SUMOylation bands compared to the WT control. Double or triple mutants, on the other hand, almost completely abolished the formation of SUMOylation. These results indicated that the K499, K576, and 624R sites all contributed to the formation of SUMO conjugates on MRTF-A, and mutation on any two sites of them would prevent SUMOylation.

It was reported that the nuclear localization of MRTF-A was associated with the transactivation of an angiogenic factor CCN1, which is an early response gene critical for vascular development and repair [28]. To further study the downstream events that may be impacted by *SENP1* knockdown and its regulation on MRTF-A SUMOylation, the expression and activity of CCN1 were analyzed. As shown in Figure 4D,E. The expression of CCN1 was in line with the level of SUMOylation of MRTF-A, which increased continuedly when suppressing SUMOylation with single, double, and triple lysine-substitution mutants. This suggests that SUMOylation of MRTF-A blocks its interaction or transactivation of CCN1. The activation of CCN1 was further analyzed by luciferase assay, which confirms that abolishing MRTF-A SUMOylation could increase the activation of CCN1 (Figure 4F). These results not only indicate that the three lysine residues, Lys-499, Lys-576, and Lys-624, are the major sites for SUMOylation in MRTF-A but also revealed the possible downstream regulation network to affect angiogenesis through MRTF-A SUMOylation.

To further confirm the functional regulation of MRTF-A by SUMO1, a conjugation-defective mutant form of SUMO1 (∆GG), which deleted the two C-terminal Gly residues that are required for isopeptide bond formation, was co-transfected with MRTF-A and shSENP1 into HAECs. As shown in Figure 4C, overexpression of SUMO1(∆GG) abolished the formation of SUMOylated MRTF (slower migration bands) as determined by western blot analysis using anti-MRTF-A antibodies. In addition, qRT-PCR and western blot analysis demonstrated that overexpression of SUMO-1 (∆GG) stimulated the expression of CCN1, which was related to the reduced SUMOylation of MRTF-A (Figure 4G). The transactivation of CCN1 by SUMO-1 (∆GG) and MRTF-A was also confirmed by the Luciferase assays (Figure 4H). Taken together, these results demonstrated the modification of MRTF-A by SUMO1 played important regulation roles for its cellular functions.

### 3.6. SENP1 Knockdown Abolished the Synergetic Induction of CCN1 by the Coordination between MRTF-A and P300

**Figure 5:**
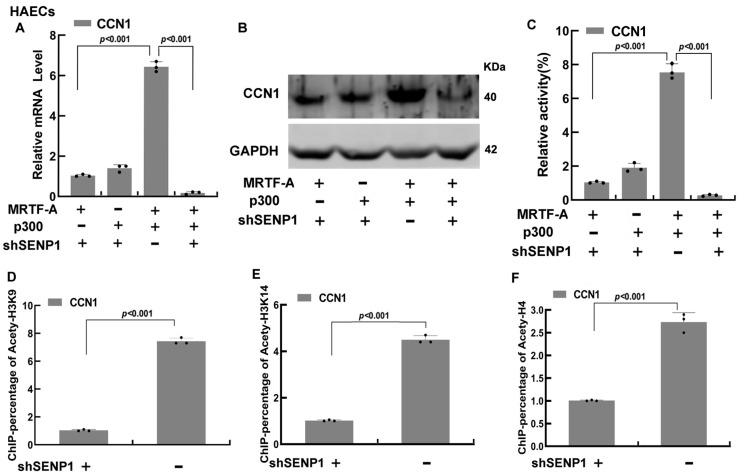
*SENP1* knockdown inhibited the acetylation of CCN1 by P300. HAECs were co-transfected with MRTF-A, P300, and shSENP1. (**A**,**B**) The expression of CCN1 were examined by qRT-PCR and Western blot. (**C**) CCN1 transactivation was analyzed using luciferase assay after 24 h transfection of MRTF-A, P300, shENP1, and CCN1 promoter-Luc plasmids into HAECs. (**D**–**F**) ChIP assays were performed in HAECs after transfection, and cross-linked chromatin was immunoprecipitated with specific anti-acetyl-h3K9/h3K14 /h4 antibodies and IgG antibody as a negative control. The signs ‘+’ means present and ‘−’ means absent for the conditions indicated in each graph. Quantitation is shown as the means ± SD (*p* values are as indicated, *n* = 3. Individual data points were shown as scattered black dots).

As a multi-function matricellular protein, the activation of CCN1 is also regulated by acetylation. Studies showed that the histone acetyltransferase p300 interacted with the C-terminal TAD domain of MRTF-A, which could synergistically enhance the expression of CCN1 [29,30]. Here, we further investigated whether SUMOylation and acetylation may interfere with each other by co-transfection of MRTF-A and P300 in HAECs. As shown in Figure 5A–C, over-expression of both MRTF-A and P300 significantly enhanced CCN1 transactivation. *SENP1* knockdown completely abolished this synergistic effect, suggesting that SUMOylation could prevent the interaction between MRTF-A and p300. Further study using ChIP assay to detect the acetylation level surrounding the SRF site of the CCN1 promoter in HAECs showed that *SENP1* knockdown inhibited histone3K9, histone3K14, and histone4 (Figure 5D–F). These results revealed an interesting signaling pathway on how defective regulation of SUMOylation may impact cell angiogenesis and mitigation.

## 4. Discussion

The angiogenic potential of MSCs plays a crucial role in maintaining vascular integrity. In the current study, we demonstrated that increased SUMOylation of MRTF-A and reduced CXCR4 expression, as a result of *SENP1* knockdown, significantly impacted MSC-mediated angiogenesis and cell migration by preventing the translocation of MRTF-A into the nucleus. We identified the angiogenic factor CCN1 as the key downstream effector following SUMOylation and revealed the abolished synergistic co-activation of CCN1 by MRTF-A and histone acetyltransferase p300 is responsible for the above impacts. These results revealed an important regulation mechanism in controlling MSC differentiation toward angiogenesis or arteriogenesis.

Previous studies have shown the importance of MRTF-A in vascular development, migration, and invasion via Rho and SRF signaling pathways [31,32,33]. However, the regulation of MRTF-A function by SUMOylation has not been addressed. Our results in the current study indicated that modulation of SUMOylation had strong effects on EC migration and angiogenesis. The identification of the three SUMO-accepting lysine sites on MRTF-A and mutation analysis of the related gene expression changes confirmed that the impact on angiogenesis was through SUMOylation of MRTF-A instead of other protein targets. Unbalanced SUMOylation is known that can result in tumour progression and other diseases [34,35,36]. Therefore, it is reasonable to assume that dysregulated SUMOylation on MRTF-A and MSC-associated angiogenesis may contribute to the occurrence of certain diseases relating to or relying on angiogenesis and vascular functions.

As one of the major SUMO-specific cysteine proteases, *SENP1* knockdown inhibited the translocation of MRTF-A into the nucleus (Figure 3), indicating that SUMOylation serves as a mechanism in holding MRTF-A in the cytoplasm in MSCs. SENP1 has been investigated in many cancers including prostate cancer, breast cancer, and colon cancer, which played important roles in suppressing cell proliferation and invasion [37,38]. The present study is consistent with these findings and provided further information that MRTF-A might be a major mediator downstream.

The present study indicated that the inhibition of angiogenesis following *SENP1* knockdown was through the impact of MRTF-A on CCN1, which was confirmed by mutation analysis through disrupting SUMO-conjugation sites. As a matricellular protein required for angiogenesis and vasculogenesis during embryonic development, the expression of CCN1 is dynamically controlled [39]. CCN1 promotes endothelial cell growth and migration partly through cell surface integrins [40]. The proangiogenic property of CCN1 has been demonstrated in different research models, which improved angiogenesis and collateral blood flow to a greater extent than VEGF [41,42]. It is worth pointing out, SENP2 may also play a role in angiogenesis. A recent study showed that SENP2 regulates the succinate dehydrogenase (SDH) complex assembly under hypoxia, which affects mitochondrial function and angiogenesis [43]. Inhibition of SENP2 has also been reported that can promote cardiac regeneration via activating the Akt pathway [44]. However, there is no evidence to suggest a direct connection with either MRTF-A or CCN1.

MRTF-A was shown to bind the CArG box within the *CCN1* promoter via SRF to stimulate the expression of CCN1 [45,46]. As to the mechanism of how MRTF-A SUMOylation affected the activity of CCNs, our data suggest that the lack of interaction between p300 and MRTF-A played a major role. The synergistic effect between MRTF-A and p300 on CCN1 expression had been demonstrated in earlier studies [29]. The current study revealed an interesting coordination between different protein modification processes since SUMOylation of MRTF-A could affect the acetylation mediated by p300.

Interestingly, the chemokine receptor CXCR4 seems to play a regulatory role on MRTF-A SUMOylation since its overexpression could partially suppress the level of the latter induced by *SENP1* knockdown (Figure 3). Together with the results that CXCR4 overexpression upregulated CCN1 level, and *SENP1* knockdown suppressed its expression, these data indicate that CXCR4 was likely to play a role upstream of MRTF-A. CXCR4 is a chemokine receptor specific for stromal cell-derived factor-1 (SDF-1), which regulates numerous activities such as chemotaxis, adhesion, and cell proliferation [47,48]. SDF-1α/CXCR4 signaling was reported previously that played a crucial role in the process of pathological neovascularization [49]. While further studies are required to know more details underlying the interactions and signaling pathways on how *SENP1* knockdown upregulated CXCR4 by what mechanism it affected MRTF-A SUMOylation, the data presented in the current study suggest that cytokines/chemokines may serve as another layer of regulation on MSC angiogenesis in addition to SENP1/SUMOylation.

**Figure 6:**
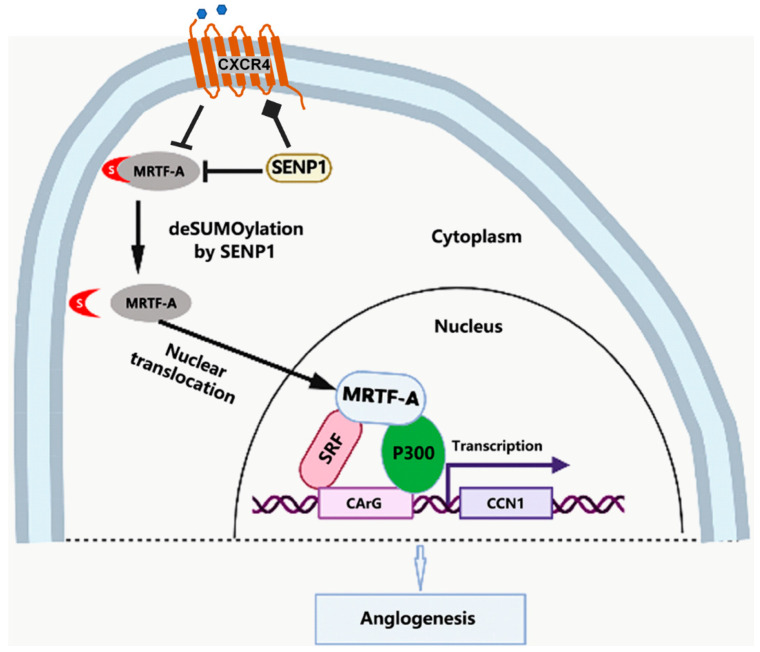
A model for the regulation of MRTF-A by SUMOylation. MRTF-A is sequestered in the cytoplasm by SUMOylation in MSCs. When there is a need for differentiation toward angiogenesis, SENP1 is activated by certain regulation factors to induce MRTF-A deSUMOylation, which makes MRTF-A relocate into the nucleus. By cooperating with P300 and SRF on the CArG box, the angiogenic factor CCN1 is transactivated and initiates the angiogenesis process. CXCR4 likely plays a regulatory role on upstream of MRTF-A SUMOylation, but the detailed interactions are yet to be elucidated.

In conclusion, the current study revealed that SENP1 knockdown led to increased MRTF-A SUMOylation, which inhibited arterial-specific endothelial angiogenesis of hMSCs via down-regulating CCN1 (a proposed signaling pathway is presented in Figure 6). The regulation of MRTF-A function by SUMOylation may potentially impact a variety of cellular processes such as revascularization, wound healing, and progression of cancer.

## Data Availability

All data are available within the article and in Supplementary Materials.

## Supplementary Materials

The following supporting information can be downloaded at: https://www.mdpi.com/article/10.3390/biomedicines11030914/s1, Table S1: Primers used for PCR; Table S2: Primers used for PCR during ChIP analysis. Figure S1: Overexpression of CXCR4 restored the cell invasion capacity; Figure S2: Knockdown of SENP1 suppressed the mRNA level of SDF-1 as determined by RT-PCR.
