# Supplementary material for: Knockdown SENP1 Suppressed the Angiogenic Potential of Mesenchymal Stem Cells by Impacting CXCR4-Regulated MRTF-A SUMOylation and CCN1 Expression
Source: Biomedicines. 2023 Mar 15;11(3):914. doi: 10.3390/biomedicines11030914 (PMC10046070; doi:10.3390/biomedicines11030914)
Supplement: Supplementary file 1 [file biomedicines-11-00914-s001.zip › biomedicines-2176175-supplementary.pdf]

**Table S1: Primers used for PCR.**

| | Forward | Reverse |
|---|---|---|
| Dll4 | CAGTGGGCAGCGAAGCTACA | ACAGGCAGTGGTAGCCATCCTC |
| Ephrin B2 | CTCCTCAACTGTGCCAAACCA | GGTTATCCAGGCCCTCCAAA |
| Notch1 | CATCACCTGCCTGTTAGGAG | ACACATGGCAACATCTAACCC |
| Hey2 | TTCAAGGCAGCTCGGTAACTGAC | CATACTGATGCACTGCTGGATGG |
| SUMO1 | CAGGAGGCAAAACCTTCAAC | CTCCATTCCCAGTTCTTTCG |
| SUMO2 | ACGAGAAACCCAAGGAAGGA | CTCCATTTCCAACTGTGCAG |
| SENP1 | TTGGCCAGAGTGCAAATGG | TCGGCTGTTTCTTGATTTTTGTAA |
| SENP2 | AGCCTGGTGGTGATTGACCTAAGA | AGCTGTTGAGGAATCTCGTGTGGT |
| CCN1 | AGCAGCGTTTCCCTTCTAC | TGAGTCCCATCACCCACA |
| GAPDH | GAGCTGAACGGGAAGCTCACTG | TGGTGCTCAGTGTAGCCCAGGA |
| shSENP1 | TCGAGCGCCAGATTGAAGAACTCGAG TTCTGTTCTTCAATCTGGCGCTTTTTG | GATCCAAAAAGCGCCAGATTGAAGAA CAGAACTCGAGTTCTGTTCTTCAATCT GGCGC |

**Table S2: Primers used for PCR during ChIP analysis.**

| | Forward | Reverse |
|---|---|---|
| CCN1 | CAGGTTGCGTAGCCATCC | TGGTAGCCACCTGCCTCT |

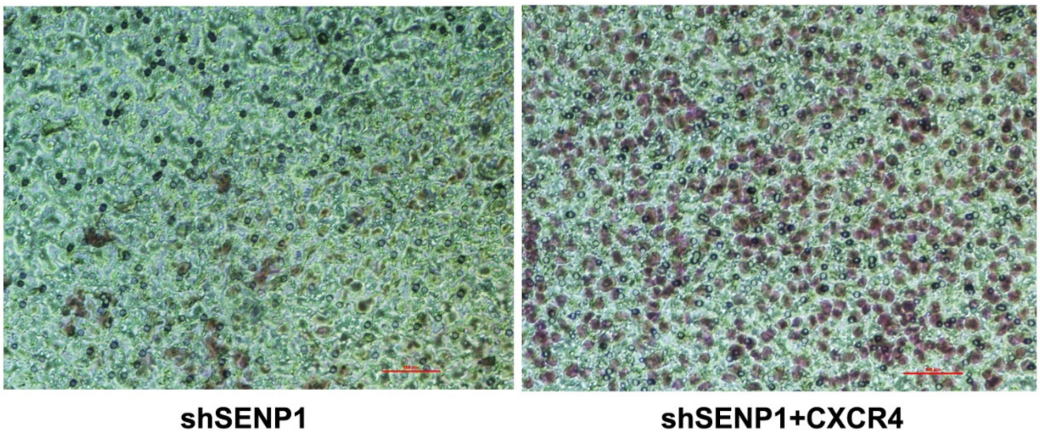


**Figure S1:** Overexpression of CXCR4 restored the cell invasion capacity. The migration capabilities of $MSCs^{shSENP1}$ group and $MSCs^{shSENP1}$ with CXCR4 overexpression group were analysed using a Transwell assay with $1.0 \times 10^4$ cells in 200 μL of a 1% FBS-containing medium. The lower chamber was filled with 600 μL of medium containing 10% FBS. Twenty-four hours later, cells that had migrated to the lower side of the membrane were fixed in 4% paraformaldehyde and stained with crystal violet.

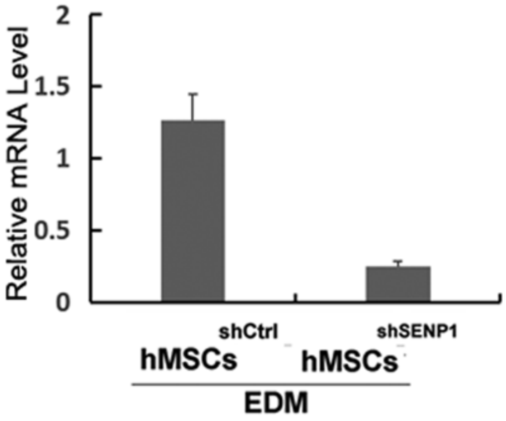


**Figure S2:** Knockdown of SENP1 suppressed the mRNA level of SDF-1 as determined by RT-PCR.
